# Establishment and characterization of two cabazitaxel-resistant prostate cancer cell lines

**DOI:** 10.18632/oncotarget.24609

**Published:** 2018-03-05

**Authors:** Kazuaki Machioka, Kouji Izumi, Yoshifumi Kadono, Hiroaki Iwamoto, Renato Naito, Tomoyuki Makino, Suguru Kadomoto, Ariunbold Natsagdorj, Evan T. Keller, Jian Zhang, Atsushi Mizokami

**Affiliations:** ^1^ Department of Integrative Cancer Therapy and Urology, Graduate School of Medical Science, Kanazawa University, Kanazawa, 920-8640, Japan; ^2^ Department of Urology, School of Medicine and Biointerfaces Institute, University of Michigan, Ann Arbor, MI, 48109, USA; ^3^ Center for Translational Medicine, Guangxi Medical University, Medical Science Research Building, Nanning, Guangxi, 530021, P. R. China

**Keywords:** prostate cancer, CRPC, cabazitaxel-resistant, MDR1

## Abstract

Once castration-resistant prostate cancer (CRPC) become resistant for cabazitaxel treatment, the patients are obliged to best supportive care. Therefore, the elucidation of the mechanism of the cabazitaxel-resistance and the conquest are important themes to improve the prognosis of the patients. Then we tried to establish cabazitaxel-resistant CRPC cell lines and characterized them. We established two cabazitaxel-resistant cell lines, PC-3-TxR/CxR and DU145-TxR/CxR from PC-3-TxR and DU145-TxR cell lines previously we established. PC-3-TxR/CxR and DU145-TxR/CxR cells became resistant for cabazitaxel by 11.8-fold and 4.4-fold, respectively. The TxR/CxR cells showed cabazitaxel-resistant using SCID mice *in vivo*. Although expression of multi-drug resistance gene 1 (MDR1) was up-regulated in DU145-TxR compared with DU145 cells, it was not up-regulated in DU145-TxR/CxR cells any more. In contrast, expression of MDR1 gene was up-regulated in PC-3-TxR compared with PC-3 cells and it was further up-regulated in PC-3-TxR/CxR compared with PC-3-TxR cells. Comparison of cDNA microarray between PC-3-TxR and PC-3-TxR/CxR cells or between DU145-TxR and DU145-TxR/CxR cells revealed that many genes were up-regulated or down-regulated. Finally, knockdown of MDR1 recovered the sensitivity to cabazitaxel not only in PC-3-TxR/CxR cells but also DU145-TxR/CxR cells. Together, regulation of MDR1 gene is important for conquest of the cabazitaxel-resistance.

## INTRODUCTION

Prostate cancer (PCa) is the most common malignancy and the second most frequent cause of cancer related death of men in the United States in 2017. There will be an estimated 161,360 new prostate cancer cases and 26,730 estimated prostate cancer-related deaths in the United States in 2017 [1]. Androgen deprivation treatment is very effective at inducing response for advanced or metastatic PCa [2, 3]. However, more than half of those cases become resistant to androgen deprivation treatment after several years [4] in what is termed castration resistant prostate cancer (CRPC). In 2012, the food and drug administration (FDA) in the United States approved the use of abiraterone and enzalutamide as effective treatments for CRPC [5, 6]. However, they eventually fail and chemotherapeutics, primarily taxanes, are initiated. The taxane docetaxel is a standard of care for CRPC and induces a high response rate for CRPC [7, 8]. However, most patients acquire resistance resulting in cancer progression. Docetaxel resistance can develop through numerous mechanisms, including androgen receptor (AR) signaling [9], activation of prosurvival pathways [10] and the acquisition of a cancer stem cell morphology [11–13]. Expression of P-glycoprotein (P-gp) also contributes to development of resistance to taxanes such as docetaxel and paclitaxel [14–16]. Once docetaxel resistance develops, the prognosis is poor for PCa patients.

In 2010, the FDA approved the use of cabazitaxel as second line treatment for these patients that developed resistance to docetaxel. Similar to docetaxel, cabazitaxel inhibits cell division by promoting polymerization of tubulin and stabilizing microtubules. The effectiveness of paclitaxel and docetaxel is limited by the high substrate affinity of both agents for P-gp, an adenosine triphosphate (ATP)-dependent drug efflux pump that decreases the intracellular concentrations of these drugs. It has been shown that cancer cells that express P-gp become resistant to taxanes. In contrast, cabazitaxel has poor affinity for P-gp, and it shows an anti-tumor effect even for PCa that are resistant to docetaxel [17]. In clinical practice, cabazitaxel has shown effectiveness for docetaxel-resistant CRPC patients [18–20]. However, once resistance to cabazitaxel is acquired, there are limited therapeutic options other than continuing symptomatic and supportive care. Thus, it is critical to understand the mechanisms through which cabazitaxel-resistance develops in PCa. We previously established paclitaxel/docetaxel-resistant PCa cells, PC-3-TxR and DU145-TxR, and characterized those cell lines previously [14, 21]. To mimic the clinical progression, we used these cell lines to develop cabazitaxel-resistant prostate cancer cell lines and characterized those cell lines.

## RESULTS

### Establishment of cabazitaxel-resistant cell lines

**Figure 1:**
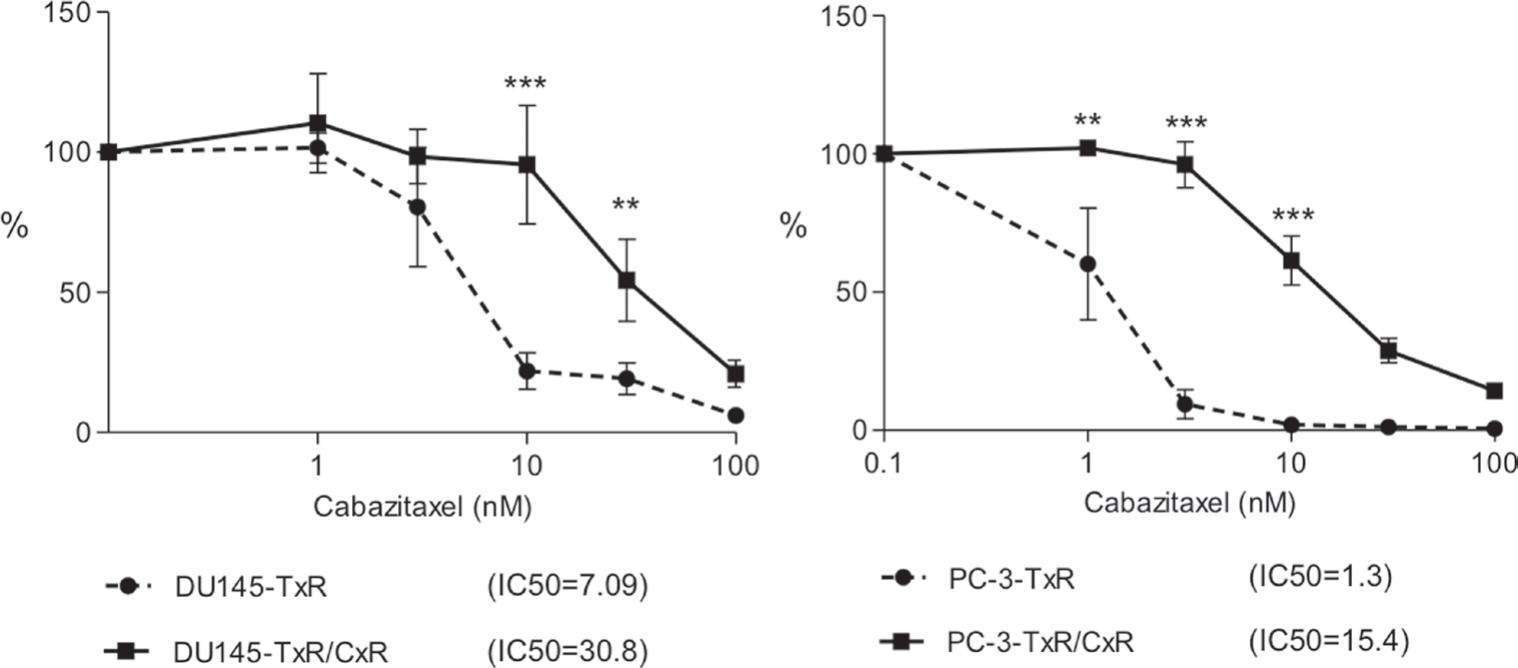
Growth inhibition by cabazitaxel DU145-TxR, DU145-TxR/CxR, cabazitaxel-resistant DU145-TxR/CxR, PC-3-TxR, and PC-3-TxR/CxR cells were exposed with indicated concentrations of cabazitaxel for 24 h and then counted 2 days after exposure. *p*-value: ^*^ < 0.05; ^**^< 0.01, and ^***^< 0.001.

We confirmed the sensitivity of cabazitaxel for PC-3 and DU145. Cabazitaxel is typically used only after docetaxel use, so we further established cabazitaxel resistant cell lines from cell lines that were resistant to docetaxel. IC_50_ of PC-3-TxR and DU145-TxR for cabazitaxel was 1.3 nM and 7.09 nM, respectively. We speculated that DU145-TxR cells demonstrated greater cabazitaxel-resistance than PC-3-TxR cells as we previously demonstrated that DU145-TxR cells had higher P-gp expression than PC-3-TxR cells [14]. Next, we tried to establish cabazitaxel-resistant cell lines using a stepwise increasing concentration of cabazitaxel (starting at 0.1 nM) in the TxR cells. It took approximately 6 months to establish cabazitaxel-resistant cells (PC-3-TxR/CxR, DU145-TxR/CxR) from the start of treatment with cabazitaxel. The IC_50_ of PC-3-TxR and PC-3-TxR/CxR cells was 1.3 nM and 15.4 nM, and the IC_50_ of DU145-TxR and DU145-TxR/CxR cells was 7.0 nM and 30.8 nM, respectively. The fold change of IC_50_ of PC-3-TxR/CxR and DU145-TxR/CxR cells compared to their respective TxR cell lines were 11.8-fold and 4.3-fold, respectively (Figure 1).

**Figure 2:**
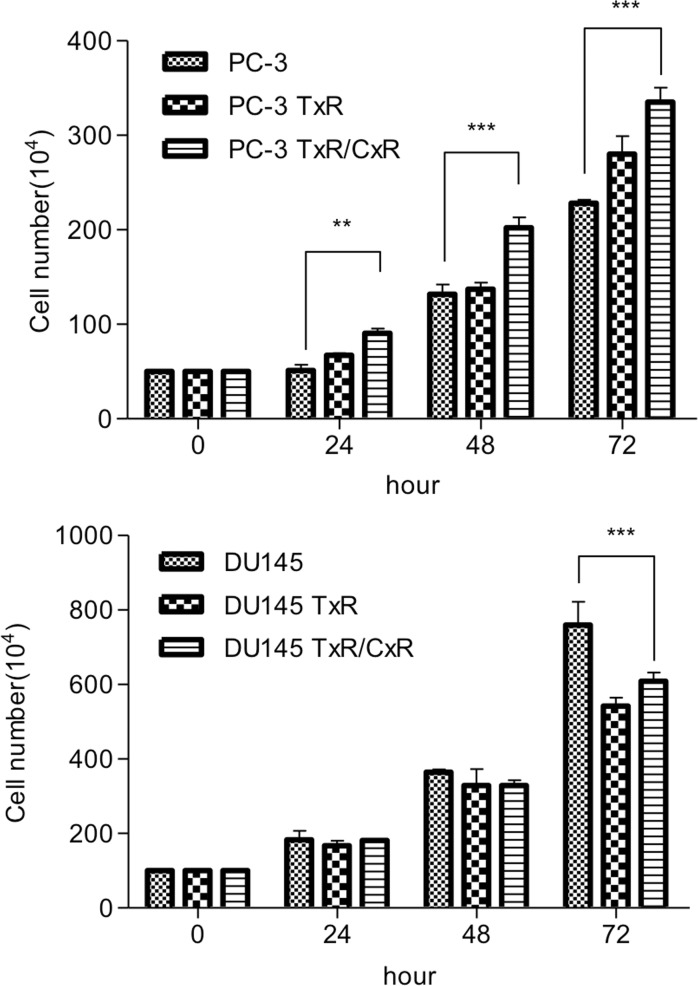
The proliferation rate of each cell lines PC-3 (wild-type, TxR, TxR/CxR) and DU145 (wild-type, TxR, TxR/CxR) cells were seeded in six well plate (PC-3: 5 × 10^5^ cells/well, DU145: 1 × 10^6^ cells/well) and were cultured for 3 days. *p*-value: ^*^< 0.05; ^**^< 0.01, and ^***^< 0.001.

We compared the proliferation of the PC-3 (parent, TxR, and TxR/CxR) and DU145 (parent, TxR, and TxR/CxR) cells *in vitro*. The proliferation rate of PC-3-TxR/CxR cells was significantly higher than that of parent PC-3 cells. In contrast, the proliferation rate of DU145-TxR and DU145-TxR/CxR cells significantly decreased compared to parent DU145 cells, although no significant difference was observed between DU145-TxR and DU145-TxR/CxR cells (Figure 2).

### Cabazitaxel-resistance *in vivo*

**Figure 3:**
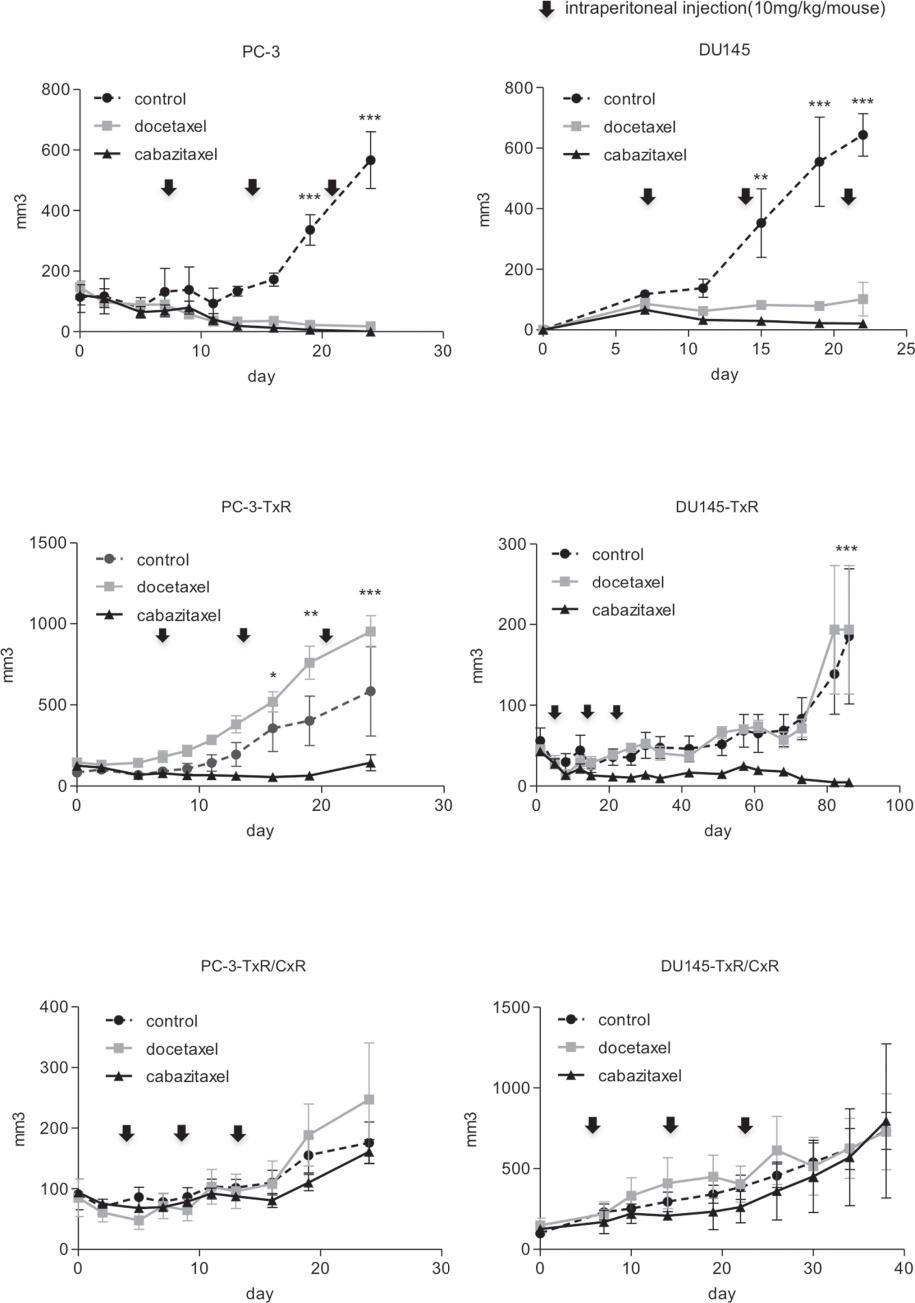
Tumor growth of cabazitaxel-resistant cell lines *in vivo* PC-3 (wild-type, TxR, TxR/CxR) and DU145 (wild-type, TxR, TxR/CxR) cells (2 × 10^6^/mouse) with 50% Matrigel were implanted into SCID mice subcutaneously. The control group was injected with 50 μL of DMSO. Docetaxel and cabazitaxel groups were injected weekly at doses of 10 mg/kg. Tumors were measured 2–3 times a week using a caliper. *p*-value: ^*^< 0.05; ^**^< 0.01, and ^***^< 0.001.

In order to confirm that the TxR/CxR cell lines show cabazitaxel-resistance *in vivo*, we implanted these cells into SCID mouse and treated with docetaxel or cabazitaxel. Although parent PC-3 and DU145 cells as control groups showed tumor growth, their growth was effectively inhibited by both docetaxel and cabazitaxel administration. In contrast, cabazitaxel effectively inhibited growth of both PC-3-TxR and DU145-TxR; whereas, docetaxel had minimum impact on their growth. Finally, neither docetaxel nor cabazitaxel significantly impacted the growth rate of PC-3-TxR/CxR and DU145-TxR/CxR cells (Figure 3). These results demonstrate that the PC-3TxR/CxR and DU145-TxR/CxR developed resistance to cabazitaxel.

### Expression of chemoresistance-related genes

**Figure 4:**
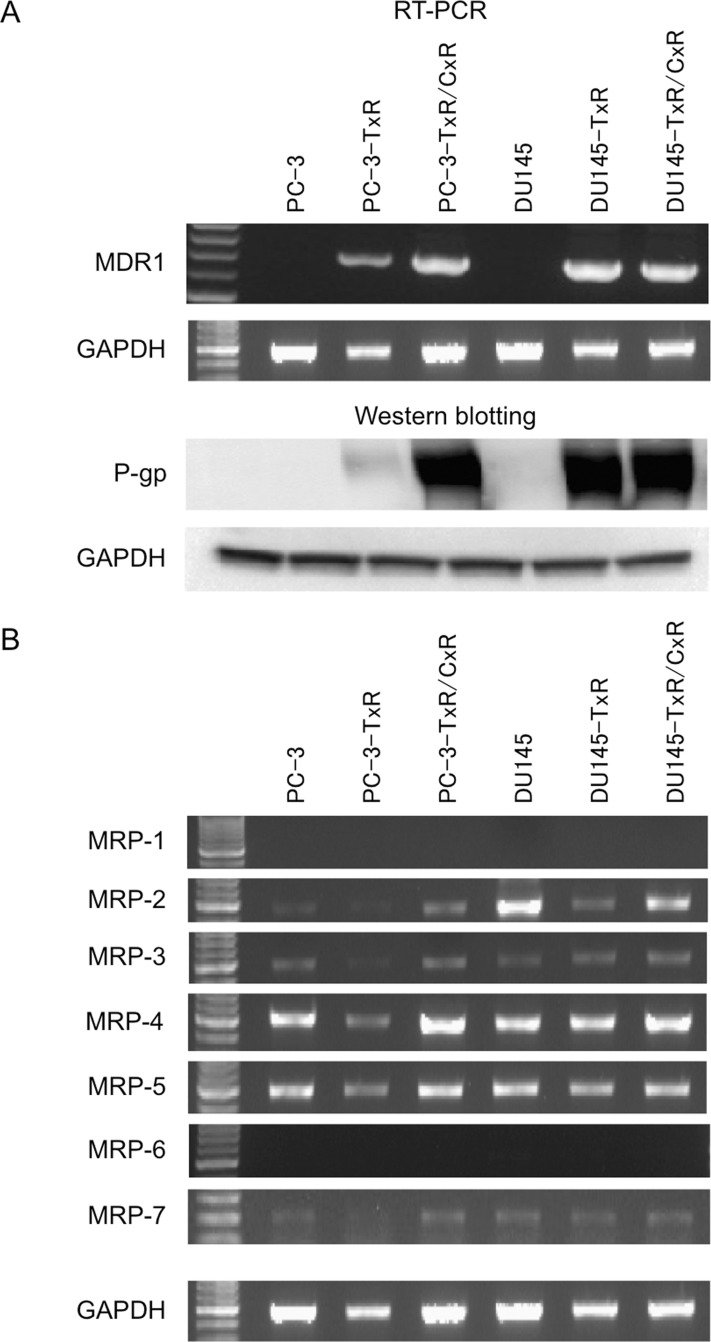
Expression of various drug resistance-related genes in parent, docetaxel-resistant, and cabazitaxel-resistant cells (**A**) RT-PCR of MDR1 and western blot analysis of P-gp in PC-3, and PC-3-TxR, PC-3-TxR/CxR, DU145, DU145 –TxR, DU145-TxR, and DU145-TxR/CxR cells. After mRNA was purified from these cells, RT-PCR of MDR1 mRNA was performed. Expression of P-gp. Whole protein was extracted as described in Materials and Methods and loaded on a SDS-polyacrylamide gel for western blot analysis. After protein was transferred to a membrane, anti-P-gp antibody and anti-GAPDH antibody were employed for detection of 170 kDa P-gp and 37 kDa GAPDH protein, respectively. (**B**) Expression of MRP1-7 genes in parent, docetaxel-resistant, and cabazitaxel-resistant cells.

Although variety of mechanisms that contribute to taxanes-resistance have been proposed, mechanisms through which cabazitaxel-resistance develops are unclear. One of the mechanisms appears to be up-regulation of genes that decrease intracellular drug concentrations through increasing drug efflux from the cells. P-gp and multidrug resistance protein (MRP), which belong to the ATP-binding cassettes (ABC) family, are well-known typical drug transporters. First, we evaluated the expression of MDR1 gene and P-gp in DU145, DU145-TxR, DU145-TxR/CxR, PC-3, PC-3-TxR, and PC-3-TxR/CxR cells (Figure 4A). Expression of MDR1 and P-gp was elevated in PC-3-TxR cells compared with parent PC-3 cells and this expression was further elevated in PC-3-TxR/CxR cells. In contrast, expression of MDR1 and P-gp in DU145-TxR cells was already highly elevated compared with parent DU145 cells, and expression of MDR1 mRNA and P-gp was not further elevated in DU145-TxR/CxR cells. Next, we evaluated the expression MRP1 through MRP7 of DU145, DU145-TxR, DU145-TxR/CxR, PC-3, PC-3-TxR, and PC-3-TxR/CxR cells by RT-PCR (Figure 4B). Only the expression level of MRP2, which was repressed in both TxR cells compared with both parent cells, was elevated in both TxR/CxR cells. The expression of MRP3, MRP4, and MRP5 in PC-TxR/CxR cells was increased compared with PC-3-TxR cells, but not DU145-TxR/CxR cells.

### cDNA microarray analysis

In order to reveal the mechanisms that contribute to the development of cabazitaxel resistance, we compared gene expression profiles among PC-3-TxR, PC-3-TxR/CxR, DU145-TxR, and DU145-TxR/CxR cells by cDNA microarray analysis. 4,470 genes in PC-3-TxR/CxR cells and 1,345 genes in DU145-TxR/CxR cells were up-regulated by more than 3-fold compared to PC-3-TxR and DU145-TxR, respectively, and 4,683 genes in PC-3-TxR/CxR cells and 1,537 genes in DU145-TxR/CxR cells were down-regulated less than 0.3-fold compared to PC-3-TxR and DU145-TxR, respectively. Consistent with our earlier findings, although expression of MDR1 gene was up-regulated in DU145-TxR cells by 500-fold compared with DU145 cells, it was up-regulated in DU145-TxR/CxR cells by 1.2-fold (Table 1). Whereas, expression of MDR1 gene was up-regulated in PC-3-TxR cells by 19-fold compared with PC-3 cells and it was further up-regulated in PC-3-TxR/CxR by 53-fold compared with PC-3-TxR cells (Table 2). The relative expression ratio of MRP1 through MRP6 was similar to the cDNA microarray analysis (Tables 1 and 2).

**Table 1:** Expression ratio of chemoresistance-related genes among series of DU145 cell lines in cDNA microarray analysis

Gene name	Normalized signal			Ratio	
	DU145	DU145-TxR	DU145-TxR/CxR	DU145-TxR:DU145	DU145-TxR/CxR:DU145-TxR
MDR1	0.024	12.629	14.701	517.36	1.16
MRP1	1.242	0.709	0.691	0.57	0.98
MRP2	7.570	0.224	1.218	0.03	5.43
MRP3	5.451	4.871	3.580	0.89	0.73
MRP4	0.371	0.450	0.472	1.21	1.05
MRP5	2.454	1.611	1.630	0.66	1.01
MRP6	0.039	0.030	0.049	0.78	1.60
MRP8	0.022	0.020	0.018	0.93	0.91
MRP9	0.024	0.023	0.021	0.93	0.92
MRP10	0.406	0.395	0.413	0.97	1.04
MRP11	0.024	0.022	0.020	0.93	0.92
MRP12	0.021	0.020	0.018	0.94	0.92
MRP13	0.024	0.022	0.020	0.92	0.92

**Table 2:** Expression ratio of chemoresistance-related genes among series of PC-3 cell lines in cDNA microarray analysis

Gene name	Normalized signal			Ratio	
	PC-3	PC-3-TxR	PC-3-TxR/CxR	PC-3-TxR:PC-3	PC-3-TxR/CxR:PC-3-TxR
MDR1	0.020	0.392	20.730	19.26	52.88
MRP1	0.786	0.630	0.801	0.80	1.27
MRP2	0.058	0.025	0.268	0.43	10.75
MRP3	1.528	1.252	4.008	0.82	3.20
MRP4	0.153	0.128	0.386	0.83	3.03
MRP5	0.232	0.324	0.795	1.40	2.45
MRP6	0.051	0.079	0.802	1.55	10.10
MRP8	0.045	0.018	0.021	0.40	1.17
MRP9	0.020	0.018	0.019	0.91	1.06
MRP10	0.446	0.584	0.407	1.31	0.70
MRP11	0.019	0.017	0.018	0.90	1.05
MRP12	0.017	0.015	0.016	0.89	1.06
MRP13	0.019	0.017	0.018	0.91	1.03

### Recovery of cabazitaxel sensitivity by MDR1 knockdown

**Figure 5:**
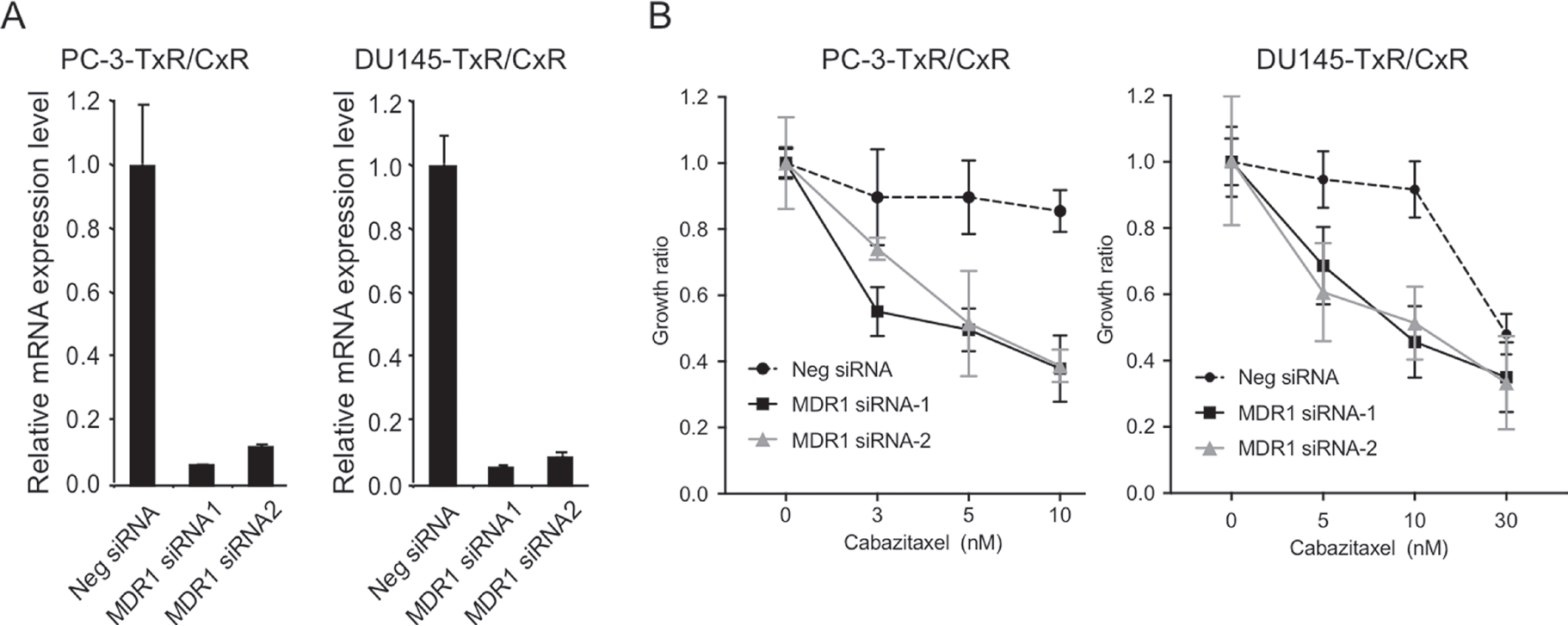
Recovery of cabazitaxel sensitivity by knockdown of MDR1 (**A**) Knockdown of MDR1 mRNA expression in cabazitaxel-resistant cells. After MDR1 expression was knockdown by transfection of 10 nM negative (Neg) siRNA, MDR1 siRNA1 or siRNA2 into PC-3-TxR/CxR and DU145-TXR/CxR cells for 24 h, total RNA was extracted and real time RT-PCR of MDR1 mRNA was performed. Relative MDR1 mRNA expression was by normalized by GAPDH expression. (**B**) Growth ratio of PC-3-TxR/CxR and DU145-TxR/CxR cells. After knockdown of MDR1 mRNA by 10 nM siRNA for 24 h, cells were treated with indicated concentrations of cabazitaxel for 24 h. Two days later, number of cells was counted.

Although overexpression of P-gp is an important factor for DU145 cells acquiring resistance to paclitaxel, overexpression of P-gp was not a main factor for PC-3 cells acquiring resistance to paclitaxel [14]. However, expression level of MDR1 and P-gp was further elevated in PC-3-TxR/CxR compared with PC-3-TxR which led us to speculate that MDR1 might contribute to cabazitaxel-resistance, especially in PC-3-TxR/CxR cells. To test this hypothesis, we proposed to assess the impact of knocking down MDR1 expression on resistance to cabazitaxel. We first confirmed that 2 different MDR1 siRNA down-regulated MDR1 expression by greater than 80% in PC-3-TxR/CxR and DU145-TxR/CxR cells (Figure 5A). Knockdown of MDR1 by MDR1 siRNA-1 and siRNA-2 recovered the sensitivity to cabazitaxel in PC-3-TxR/CxR cells (Figure 5B). Interestingly, knockdown of MDR1 by MDR1 siRNA-1 and siRNA-2 also recovered the sensitivity to cabazitaxel in DU145-TxR/CxR cells.

## DISCUSSION

The observation that cabazitaxel is effective in CRPC patients that have developed docetaxel resistance suggests that mechanisms, other than those that account for docetaxel resistance, contribute to the development of cabazitaxel resistance after failure of docetaxel treatment. In order to facilitate defining mechanisms that promote cabazitaxel resistance in CRPC patients after docetaxel treatment, we established two cabazitaxel-resistant cell lines, PC-3-TxR/CxR and DU145-TxR/CxR from the paclitaxel-resistant cell lines, PC-3-TxR and DU145-TxR cell lines, respectively. While PC-3-TxR and DU145-TxR cells were resistant to docetaxel, they were sensitive to cabazitaxel, In contrast, PC-3-TxR/CxR and DU145-TxR/CxR cells showed higher resistance to cabazitaxel than TxR cells *in vitro* and *in vivo*. Similar to docetaxel, cabazitaxel promotes polymerization of tubulin and inhibits cell division by stabilizing microtubule, but the affinity of cabazitaxel substrate to P-gp is weaker than docetaxel. Moreover, the mechanisms by which parent PC-3 and DU145 cells become paclitaxel-resistant were different between PC-3-TxR and DU145-TxR cells. The main mechanism in DU145-TxR cells was overexpression of P-gp encoded from MDR1 mRNA [14]. Knockdown of MDR1 by MDR1 siRNA recovered paclitaxel-resistance in DU-145-TxR cells. In contrast, overexpression of P-gp did not contribute to paclitaxel-resistance in PC-3-TxR cells although it was observed. Repression of C-terminal tensin like protein (CTEN) expression and inhibition of latexin expression appear to be some mechanisms through which PC-3-TxR cells became paclitaxel-resistant [21] [22]. Therefore, we speculated that different mechanisms might promote cabazitaxel-resistance in PC-3-TxR versus DU145-TxR cells.

Contrary to our expectations, overexpression of P-gp encoded from MDR1 (ABCB1) caused cabazitaxel-resistance in PC-3-TxR/CxR and DU145-TxR/CxR cells in the present study. Increased MDR1 expression was observed in PC-3-TxR/CxR cells (20-fold) compared with PC-3-TxR cells. However, expression of MDR1 mRNA and P-gp was already saturated in DU145-TxR cells and the expression in DU145-TxR/CxR cells was almost same as in DU145-TxR cells. We carried out knockdown of MDR1 in PC-3-TxR/CxR cells and recovered the sensitivity to cabazitaxel. Interestingly, knockdown of MDR1 in DU145-TxR/CxR cells also recovered the sensitivity to cabazitaxel. Lombard *et al*. demonstrated that MDR1 (ABCB1) also mediated cabazitaxel-docetaxel cross-resistance in advanced PCa [21]. Together, we speculated that overexpression of MDR1 was essential to cabazitaxel-resistance and that various genes are involved in further cabazitaxel-resistance. Therefore, knockdown of MDR1 might recover sensitivity to cabazitaxel.

Ramachandran *et al.* established a docetaxel and cabazitaxel resistant cell line, DU145-10DRCR from a docetaxel-resistant cell line, DU145-10DR using a method similar to ours and compared the expression levels in DU145-10DR and DU145-10DRCR cells using cDNA microarray [23]. They reported that DU145 acquired resistance to docetaxel by inhibiting growth arrest and DNA damage inducible alpha (GADD45a) by DNA methylation [24] and they also demonstrated that demethylation using azacytidine restored docetaxel resistance. In contrast to these previous studies, the expression level of GADD45a were not up-regulated in the cells used in the current study (0.89-fold difference between DU145-TxR and DU145-TxR/CxR cells, data not shown). Kosaka *et al*. demonstrated that cytotoxicity induced by cabazitaxel in CRPC cells using LNCaP subline caused reactive oxygen species (ROS) production. However, mRNA level of those ROS-associated species, MKK, MKK4, ELK1, and MEF2C were not significantly changed in PC-3-TxR/CxR and DU145-TxR/CxR cells based on our cDNA microarray analysis, suggesting that cabazitaxel-resistant cells may lose responsiveness for ROS [25].

It remains unknown why MDR1 is up-regulated in PC-3-TxR/CxR cells compared to PC-3-TxR cells. Demethylation of MDR1 promoter in DU145-TxR cells coincides with increased MDR1 expression in those cells but not in PC-3-TxR cells [14]. Nuclear translocation of Y-box-binding protein 1 (YB-1) was also related with overexpression of MDR1 [14, 26]. Epithelial growth factor (EGFR) mediated docetaxel-resistance through Akt-dependent expression of MDR1 [27]. MDR1 expression was also increased by introducing PTOV1 into cell lines of PC-3 and DU145 [28]. As there may be several mechanisms through which P-gp expression is regulated further investigations are necessary to determine the mechanisms through which MDR1 overexpression occurs in PC-3-TxR/CxR cells.

In addition to P-gp, the cDNA microarray analysis revealed several genes might be involved in cabazitaxel-resistance. The gene expression profile of PC-3-TxR/CxR cells was dramatically changed compared with PC-3-TxR cells suggesting that these genes are associated with cabazitaxel-resistance and may promote resistance. MRP2 was also up-regulated in PC-3-TxR/CxR and DU145-TxR/CxR cells compared with (Figure 4). Expression of MRP2, however, was down-regulated in DU145-TxR cells compared with both parent cells. Since parent PC-3 and DU145 cells were more sensitive to cabazitaxel than both TxR cells (data not shown), we speculated that MRP2 was not associated with cabazitaxel-resistance.

We hypothesize that the genes whose expression changes in both PC-3-TxR/CxR and DU145-TxR/CxR cells are likely to contribute to cabazitaxel-resistance (Table 3). Although we tried to knockdown tumor-associated calcium signal transducer 2 (TACSTD2) in TxR/CxR cells, we could not observe recovery of cabazitaxel-sensitivity (data not shown). We are currently investigating for other genes identified by the cDNA array for their role in cabazitaxel resistance.

**Table 3:** The genes which changed commonly between DU145-TxR/CxR and PC-3-TxR/CxR cells

	Up-regulated genes		DU145-TxR	DU145-TxR/CxR	Fold Change	PC3-TxR	PC3-TxR/CxR	Fold Change
Gene Name	Systematic Name	Description	Normalized	Normalized	TxR/CxR vs TxR	Normalized	Normalized	TxR/CxR vs TxR
KRTAP2-3	NM_001165252	keratin associated protein 2–3	0.07	2.84	41.4	0.03	1.44	42.2
BAIAP2L2	NM_025045	BAI1-associated protein 2-like 2	0.57	4.19	7.4	0.11	3.54	33.0
TACSTD2	NM_002353	tumor-associated calcium signal transducer 2	3.03	17.02	5.6	1.78	16.88	9.5
AP1M2	NM_005498	adaptor-related protein complex 1, mu 2 subunit	0.92	4.47	4.9	0.06	5.85	102.6
HSD17B7	NM_016371	hydroxysteroid (17-beta) dehydrogenase 7	1.36	5.43	4.0	1.68	3.85	2.3
PTPLA	NM_014241	protein tyrosine phosphatase-like, member A	3.08	8.88	2.9	0.10	5.01	50.7
CTGF	NM_001901	connective tissue growth factor	1.79	4.48	2.5	1.37	2.78	2.0
CRIP1	NM_001311	cysteine-rich protein 1	7.02	15.51	2.2	2.63	28.92	11.0
LIMA1	NM_016357	LIM domain and actin binding 1	8.58	18.29	2.1	6.42	25.78	4.0
ATP8B1	NM_005603	ATPase, aminophospholipid transporter, class I, type 8B, member 1	1.80	3.71	2.1	0.59	6.47	11.0
MYL9	NM_181526	myosin, light chain 9, regulatory	10.80	22.14	2.1	0.15	23.89	161.2
	**Down-regulated genes**		**DU145-TxR**	**DU145-TxR/CxR**	**Fold Change**	**PC3-TxR**	**PC3-TxR/CxR**	**Fold Change**
**GeneName**	**Systematic** **Name**	**Description**	**Normalized**	**Normalized**	**TxR/CxR vs TxR**	**Normalized**	**Normalized**	**TxR/CxR vs TxR**
CXCL1	NM_001511	chemokine (C-X-C motif) ligand 1	14.89	2.96	0.20	17.01	0.26	0.02
DDIT4	NM_019058	DNA-damage-inducible transcript 4	11.02	3.31	0.30	31.79	2.89	0.09

CRPC may be transformed into higher grade neuroendocrine tumor (NET) during chemotherapy [29, 30]. One of mechanisms of docetaxel-resistance and cabazitaxel-resistance may emergence of NET. We confirmed the expression of NET-related markers, chromogranin A (CGa) and nneuron-specific enolase (NSE) using cDNA microarray data [31], normalized expression of CGa was extremely low in all cell lines, and normalized signal level of NSE was 2.9, 5.6, and 0.51 in PC-3, PC-3-TxR, and PC-3-TxR/CxR and was 1.3, 0.5, and 0.45 in DU145, DU145-TxR, and DU145-TxR/CxR, respectively. cDNA microarray data suggested that these cell lines might not be related with NET.

In conclusion, we established two cabazitaxel-resistant CRPC cell lines. Up-regulation of P-gp expression was a key mediator of cabazitaxel resistance in PC-3 and DU145 cells. Based on cDNA array analysis there may be additional genes that contribute to development of resistance. Further exploration is needed to identify these. The cabazitaxel-resistant cells are a very useful tool for exploring for mechanisms of cabazitaxel-resistance and treatment strategies for CRPC in the future.

## MATERIALS AND METHODS

### Cell culture and cell proliferation assay

DU145 and PC-3 cells purchased from American type culture collection were cultured in Dulbecco’s modified Eagle medium (DMEM) and RPMI1640 containing 5% fetal calf serum (FCS) and penicillin/ streptomycin (Invitrogen, CA, USA) in a humidified incubator at 37° C with 5% CO_2_. Cell growth inhibition assay was performed by plating 1 × 10^5^ cells on 6-well plates. One day after plated, cells were treated with the indicated concentration of anticancer agents for 24 h. Then the medium was changed to normal medium and cells were cultured for 2 days. At the end of the culture period, the cells were trypsinized and counted with a hemocytometer.

### Establishment of cabazitaxel-resistant DU145 and PC-3 cell lines

To establish cabazitaxel-resistant cell lines, we used paclitaxel/docetaxel-resistant (PC-3-TxR and DU145-TxR) cancer cells. Cabazitaxel-resistant cells were established by stepwise increase of concentrations of cabazitaxel in a fashion similar to how we previously established paclitaxel resistant PC-3-TxR and DU145-TxR cells [14]. Briefly, we confirmed that the cells proliferated without death at 0.1 nM cabazitaxel. Then the concentration of cabazitaxel was increased stepwise (0.1–0.3 nM on each passage), and the cell line was established to grow in the presence of 1 nM cabazitaxel. We then further continued to process to further increase resistance. For maintenance of cabazitaxel-resistant cells, 1 nM cabazitaxel was always added into the normal medium.

### RNA extraction and RT-PCR

Forty-eight hours after plating of 4 × 10^5^ DU145 or PC-3 cells, total RNA was purified with RNeasy mini kit (Qiagen, Maryland, USA). Complementary DNA (cDNA) was made by reverse-transcription (RT) of 1 μg each total RNA using ThermoScript RT-PCR system (Invitrogen). Each cDNA sample was amplified with ExTaq (Takara, Japan). Each of the amplified PCR products was identified using electrophoresis on an 1.5% agarose gel. Real-time PCR analysis (CFX Connect™ Real-Time PCR Detection System, BIO-RAD, CA USA) was also performed to quantitate RNA level. Amplified PCR products were normalized using glyceraldehyde-3-Phosphate dehydrogenase (GAPDH). The primers used for RT-PCR were described previously [14].

### cDNA microarray analysis

Forty-eight hours after plating of 4 × 10^5^ DU145 or PC-3 cells, total RNA was purified with RNeasy mini kit. RNA samples were sent to Takara (Takara, Otsu, Japan) and were analyzed using Agilent technologies (human 8 × 60 k ver. 2).

### Small interfering RNA transfection

Multi-drug resistance gene (MDR1) (ATP-binding Cassette Sub-family B Member 1, ABCB1) small interfering RNA (MDR1 siRNA-1(HSS107918) and −2(HSS107919)) and non-targeting siRNA (Neg siRNA, 12935–400) were purchased from Invitrogen. After 3 × 10^5^ DU145-TxR/CxR and PC-3-TxR/CxR cells were cultured on 6-well plates, cells were transfected with 10 nM MDR1 siRNA1, siRNA2, or Neg siRNA by Lipofectamine^®^ RNAiMAX Transfection Reagent (Invitrogen). Twenty-four h later total RNA was extracted and quantitated by real-time PCR. In order to see the effect of MDR1 siRNA on cabazitaxel resistance, 3 × 10^5^ DU145-TxR/CxR and 5 × 10^5^ PC-3-TxR/CxR cells were transfected with 10 nM MDR1 siRNA-1, −2, or Neg siRNA after seeding on 6-well plates. Twenty-four h later cells were treated with indicated concentration of cabazitaxel for 24 h. Then the cells were cultured for 2 days and counted with a hemocytometer.

### Xenograft studies in mice

Intact male 6–7-week-old severe combined immunodeficient (SCID) mice were obtained from Clea Japan (Tokyo, Japan). 2 × 10^6^ PC-3 (wild-type, TxR/CxR) and DU145 (wild-type, TxR/CxR) cells were implanted with 50% Matrigel subcutaneously to SCID mice. When the tumor became detectable, docetaxel or cabazitaxel administrate was initiated via intraperitoneal injection. The control group was injected with 50 μL of DMSO. Docetaxel and cabazitaxel groups were injected weekly at doses of 10 mg/kg. Tumors were measured 2–3 times a week using a caliper. This animal protocol was approved by the Institutional Animal Care and Use Committee of the Graduate School of Medical Science, Kanazawa University.
